# A Survey on the Impact of Intelligent Surfaces in the Terahertz Communication Channel Models

**DOI:** 10.3390/s24010033

**Published:** 2023-12-20

**Authors:** Jefferson D. S. E Silva, Jéssica A. P. Ribeiro, Vignon F. Adanvo, Samuel B. Mafra, Luciano L. Mendes, Yonghui Li, Rausley A. A. de Souza

**Affiliations:** 1 National Institute of Telecommunications (INATEL), Santa Rita do Sapucaí 37540-000, Brazil; jefferson.david@dtel.inatel.br (J.D.S.E.S.); jessica.abranches@dtel.inatel.br (J.A.P.R.); vignon.fidele@dtel.inatel.br (V.F.A.); samuelbmafra@inatel.br (S.B.M.); lucianol@inatel.br (L.L.M.); 2 School of Electrical and Information Engineering, University of Sydney, Sydney, NSW 2006, Australia; yonghui.li@sydney.edu.au

**Keywords:** terahertz band, intelligent surface, reconfigurable intelligent surface, large intelligent surface, 5G, 6G

## Abstract

Terahertz (THz) band will play an important role in enabling sixth generation (6G) envisioned applications. Compared with lower frequency signals, THz waves are severely attenuated by the atmosphere temperature, pressure, and humidity. Thus, designing a THz communication system must take into account how to circumvent or diminish those issues to achieve a sufficient quality of service. Different solutions are being analyzed: intelligent communication environments, ubiquitous artificial intelligence, extensive network automation, and dynamic spectrum access, among others. This survey focuses on the benefits of integrating intelligent surfaces (ISs) and THz communication systems by providing an overview of IS in wireless communications with the scanning of the recent developments, a description of the architecture, and an explanation of the operation. The survey also covers THz channel models, differentiating them based on deterministic and statistical channel modeling. The IS-aided THz channels are elucidated at the end of the survey. Finally, discussions and research directions are given to help enrich the IS field of research and guide the reader through open issues.

## 1. Introduction

The fifth generation (5G) wireless network technology is already commercialized worldwide. The study groups and partnership projects have been designing the 5G standard since the last decade. About the key performance indicators (KPIs) set for 5G, it can be mentioned peak data rate of 20 Gbps, peak spectral efficiency of 30 bps/Hz, maximum channel bandwidth of 1 GHz, area traffic capacity of $10\;{\mathrm{Mbps}/m}^{2}$, end-to-end latency of 1 ms, a packet error rate of $10^{-5}$, and mobility of 500 km/h [1]. This set has been chosen to support the following new applications: virtual and augmented reality (VAR), e-commerce, Internet of things (IoT), and enhanced mobile broadband. Compared to the fourth generation (4G), 5G has expanded the mobile broadband and added support to IoT [2]. As a continuous evolution, the next step in wireless network technology, the sixth generation (6G), aims to at least double 5G KPIs on its deployment after 2030 [1,2].

In order to achieve 6G requirements, research groups have pointed out some solutions: terahertz (THz) band communications, intelligent communication environments, ubiquitous artificial intelligence (AI), extensive network automation, dynamic spectrum access, cell-free (CF) massive multiple-input multiple-output (mMIMO), Internet of space things (IoST), millimeter wave (mmWave) band communications, ambient backscatter communications, space-air-ground-sea integrated networks, and built-in network security [1,2]. THz band communications deserve special attention among the other solutions due to its overarching support. THz communication possesses enough spectrum bandwidth from 0.1 THz to 10 THz. It can achieve data rates from hundreds of Gbps to several Tbps, enabling new spectrum access, connecting IoST, and aiding network automation [3,4].

In order to understand better the contributions of THz communications, it can be important to mention some 6G envisioned applications that THz communications will support. Home wireless networks will exhibit Super Hi-Vision digital video format with a 7680×4320 resolution by sending data at a rate higher than 24 Gbps [5]. The wireless cognition function is able to control the transmission over a communication link between a device and a base station with enough bandwidth and data rate to work as an edge server to carry complex tasks in real-time computations that encompass contextual awareness, vision, and perception [4]. THz over fiber system is proposed to connect central stations and distributed THz wireless antennas to support high-resolution mobile multimedia services, wireless video distribution systems, and wireless local area networks (WLANs) [6]. THz will also contribute to ultra-high-speed small cell systems, create secure wireless communication for military and defense applications, and enable advanced health monitoring and chemical attack prevention systems [7]. Applications other than telecommunications can also be addressed using the following THz characteristics: lower single-photon energy than X-rays, high spectral resolution, and power penetration through non-metallic or non-polar materials [8].

However, the use of THz communication is still challenging in terms of hardware and environment. Electromagnetic waves are more or less attenuated at traversing the environment according to their frequency and the atmosphere temperature, pressure, and humidity. Experiments have shown that THz waves can suffer an attenuation from 10 to 10,000 dB/km in standard atmospheric conditions [9]. Water vapor absorbs and refracts THz waves significantly to separate a single THz pulse into two overlapping pulses after 1000 m of propagation [10]. Human-made structures also impose an excess loss on THz communications.

Wireless communication systems seek the most negligible absorption of electromagnetic waves as they reach an obstacle. Two waves originate from the incident wave at the occasion: reflected and refracted. The thicker the obstacle is regarding the wavelength, the more the incident wave is absorbed. In [11], two 2.92 mm thick glass layers with 1.96 mm air in between were analyzed to absorb 500 GHz waves by an absorption coefficient of 15 and a refractive index of almost 2.6. THz communications will experiment with multiple reflections in non-line of sight (NLoS) propagation, causing significant variations around the local average power. 6G systems must take those phenomena into account to be able to capture the arriving THz signal. In addition, solid-state circuits, antennas, and beamforming and beamtracking techniques still need to be enhanced to be cost-efficient and energy-efficient [12].

To overcome the challenges mentioned earlier, one of the enabling THz communication technologies being considered is the reconfigurable intelligent surface (RIS). This is essential to smart radio environment (SRE), which signifies a wireless environment that is programmable and controllable. RISs are generally defined as an intelligent thin composite material sheet that can cover walls, ceilings, buildings, and other structures capable of modifying radio waves impinging on it in a configurable way by means of external stimuli [13]. They are compared to mMIMO technology, except they can alter the channels’ characteristics between the transmitter and receiver. RIS can be used to create NLoS links in dead zone coverage areas, steer signals towards specific directions, suppress interference, build desired destructive interactions, increase channel capacity, enhance focus, uplift radio localization, recharge sensors, and recycle radio waves [13,14].

The authors of [15] showed that RIS can help wireless systems reduce active antenna chains while maintaining performance. In [16], the authors employed RIS for a downlink multi-user communication from a multi-antenna base station that provides up to 300% higher energy efficiency than regular multi-antenna amplify-and-forward relaying. An optimum phase shift that provided spectral efficiency degradation of less than 1 bps/Hz with a 2-bit quantizer was designed for a RIS-assisted large-scale antenna system in [17]. The authors in [18] utilized RIS in a multi-cell communication system to mitigate inter-cell interference and have studied the optimum location of RISs inside a four-cell communication scenario. Finally, RISs were exploited in [19] to provide additional communication links to increase both the signal-to-noise ratio (SNR) for legitimate receivers while suppressing the SNR at eavesdroppers.

### 1.1. Related Works

Due to their attractive ultra-wideband capability, THz communications have recently been widely accepted as a promising technology for next-generation optical wireless communication systems. In addition, numerous fields of application have been studied in the literature, such as agriculture [20], IoT communication [21], and space communication [22], mainly because of the progressive development of devices that support this band. In this context, THz channel modeling is paramount for meeting future network demand.

One of the difficulties for THz communication that has been found in the literature is the short range of the THz wave, leading to a limited coverage area of this band [23]. In addition, it suffers from high attenuation in the path, and its waves are highly directive, which hinders the reception of the transmitted signal [12]. This communication may be unfeasible if it adds to the ease of blockage by interior obstacles such as walls and doors [24] for this band. To overcome these limitations, different techniques using large-scale antenna arrays [25] and cooperative communication [26] have been proposed. These techniques have been widely studied to improve throughput and achieve multiple diversity. However, despite their exciting results, these techniques are complex and require costly hardware. For this reason, intelligent surfaces (ISs) have been proposed as a low-cost technique to extend signal coverage in THz communications [12,14,27].

Several surveys, magazines, and review works concern IS systems. They mainly discuss structure designs or address specific application areas. Remarkably, within the context of IS-aided THz communication channels, there have been developments in the modeling of IS unit elements and IS boosting wireless signal propagation. Table 1 summarizes the survey and review papers involving aspects of the IS-aided THz channel, as well as the recent papers on the THz communication and IS areas, which focus on design and feasibility.

It is possible to realize that different terminologies have been interchangeably used to identify an IS in the literature, namely, LIS, RIS, and IRS. Each terminology emphasizes one particular feature of the intelligent surface [27]. These terms have similar meanings for different authors. In this subsection, the term that the authors adopted for their works is used. In the subsequent sections, they refer to intelligent surfaces generically by IS. In [55,56], LIS is described as sizeable active antenna arrays, like a much larger mMIMO [57,58] comprising hundreds of antennas, which can enlarge the coverage and improve the positioning performance, which is suitable for THz scenarios. The term RIS has been commonly used to highlight the surface reconfiguration property for the incident signal. In [28], RISs were applied as anomalous reflectors and single-RF transmitters to analyze and compare the bit error probability offered by both implementations. The authors in [14] considered IRS a planar surface of many low-cost passive reflecting elements able to reshape the wireless propagation environment via software-controlled reflection. Each reflector can induce an amplitude and/or phase change to the incident signal independently. Thus, enhancing wireless communication performance by modifying the wireless channel in a so-called intelligent and programmable wireless environment.

The use of IS can benefit the THz network in different aspects and improve the quality of communication and services. IS is a technology that enables the control of channel multipath, shadowing, and path loss. These phenomena cause the fading to which the signal is subjected in a wireless communication system. They randomly introduce constructive and destructive interference, delay, and phase shift in the propagated signal. To control the effects of the environment on the communication channel, an IS performs reflection and precise steering of the signal. In order to evaluate the RIS performance, the authors in [29] studied deterministic path loss and its dependency on the operating frequency, transmission distance, relative humidity, air temperature, and pressure.

Curiously, the work in [30] proposed an IRS to control the propagation direction of the THz beam. The idea has been implemented through an efficient Taylor expansion-assisted gradient descent (TE-GD) scheme by dynamically updating the step size in the iterative process. Improved coverage performance has been achieved by intelligently adjusting the phase changes of the reflective elements. Unlike [30], in [31], an analytical model of path loss for RIS-assisted THz wireless systems was presented. The formalized proposal showed in detail the parameters that affect the molecular absorption and, consequently, the communication in THz. These parameters can be the transmission frequencies from the access point (AP) to the RIS center and from the RIS center to the users. Since the MIMO technique was essential for meeting the 6G requirements, in [32], the authors performed an IRS-assisted MIMO study considering a spherical wave channel modeling. The model regarding power gain and energy efficiency (EE) has been analyzed. It has been shown that the IRS can significantly improve the MIMO EE when operating in the near-field. An investigation of the channel estimation and transmission solutions for a mMIMO IRS-assisted THz system was conducted in [33]. The channel estimation was realized by beam training, and the quantization error was analyzed to evaluate performance.

To overcome some communication problems in the THz band, it is worth mentioning that CSI can improve the transmitter decision-making and optimize the transmission rate. Some authors have considered a perfect knowledge of the CSI, which is not observed in practice. In contrast, in [34], the authors proposed a channel estimation scheme based on compressed sensing (CS). Essentially, it purposely performed subspace search iterative atom pruning-based subspace pursuit (IAP-SP) for the acquisition of CSI. Channel recovery performance and the level of complexity compared to conventional models were analyzed. This proposal reduced up to 99.51% of the complexity level compared to the conventional ones. Different from [34], in [35], in addition to exploring channel estimation, it has maximized the data rate in a IRS-assisted THz MIMO system. Similarly, the proposed IAP-SP algorithm reduced computational complexity while maintaining accurate channel recovery. The optimal data rate of the proposed system was found at the cost of an extremely high computational load. A new deep neural network (DNN) scheme based on the fully connected feed-forward structure has achieved near-optimal communication rate performance and greatly reduced the computational complexity of the system.

Beyond the good results obtained by the authors mentioned previously, the link outage probability (OP) is one of the metrics to assess performance. Interestingly, the authors in [59] proposed a new swarm intelligence-based method to optimize phase changes in RIS elements under discrete constraints. The proposed solution was analyzed in terms of the OP. RIS can generate a significant performance gain for the THz communication system. The large number of reflecting elements at the IRS and its passive operation represent essential challenges in beamforming design for THz due to its dependence on the accuracy of the estimated CSI, considering various degrees of CSI availability, namely imperfect CSI, partial CSI, and statistical CSI [36]. The beamforming design must be varied to minimize interference and enable robust communication [37]. However, the beamforming solution depends upon the application. For instance, when designing the beamforming for vehicular communication, the goal is to reduce latency and not to increase throughput [60].

In [38], the authors exploited a secure communication for IRS-assisted THz systems. One method is based on semidefinite programming (SDP) and another on block coordinate descent (BCD). They were proposed to design the reflective matrix to maximize the secrecy rate of the system. The secrecy rate is the maximum transmission rate at which the eavesdropper cannot decode any information [61]. No matter the spy’s location, this scheme can improve the performance of the secrecy rate. Additionally, the authors in [39] have the beamforming concept and the IRS with improving the security of THz communication in a multi-input single-output (MISO) scheme. This proposal achieved better secrecy rate performance than the existing IRS-assisted design and the traditional non-IRS-assisted secure design. Furthermore, the attributes of RIS that differentiate them from other technologies have been collected in [40], outlining security and privacy attacks in RIS-assisted 6G applications.

Recently, research works attempted to clarify the modeling of the path loss of RISs, with the development of sufficiently accurate models for the power received at a given location in space when waves illuminating a RIS have been transmitted. In [41], the authors employed antenna theory to study the path loss of RISs in the far-field and proved that a RIS is capable of acting as an anomalous mirror under far-field propagation. The authors in [62] proved that the performance of each configuration can be different and needs to be judged based on the complexity and the amount of environmental information needed for a proper operation. They used an analytical approach based on the diffraction theory and the Huygens–Fresnel principle to model the path loss in both the near and far fields of RISs. Using the stationary phase method, the authors found the regimes under which the path loss depends on the sum and the product of the distances between the transmitter and the RIS, and the RIS and the receiver.

In [42], the authors suggested the use of the principle of spatial modulation for LIS data by modulating the information onto the on/off states of the LIS reflecting elements. In addition, passive beamforming was achieved by adjusting the phase shifts of the activated reflecting elements. In passive beamforming, the authors formulated an optimization problem of maximizing the average receiver SNR and solved it using the semi-definite relaxation technique. To retrieve the information data from the transmitter and the LIS, they developed a two-step detection algorithm based on compressed sensing and matrix factorization. At last, the trade-off between the passive beamforming gain and the information rate provided by RISs was investigated.

ISs are progressively impacting the evolution of wireless system architecture, access technologies, and networking protocols by transforming the wireless communication environment from naturally passive to intelligent. While extensive research efforts continue towards IS, the European Telecommunications Standards Institute (ETSI), which produces globally applicable standards for information and communications technology (ICT)-enabled systems and services, has created a new industry specification group (ISG) on RIS (ISG RIS), to review and establish global standardization for RIS technology [63]. The ETSI ISG RIS aims to identify and address some technical challenges that need to be adequately solved before the RIS can be adopted in future communication standards.

The propagation channel in IS-aided communication systems at the THz band has not been sufficiently explored in the literature, despite the IS applications have being able to mitigate the signal restrictions caused by the frequency selectivity of the THz communication channel. Most existing surveys and review papers have focused on summarizing various possible applications of IS in wireless communication [1,2,3,28,43,44,45,46]. This paper emphasizes ISs operating at the THz band with the potential to impact THz communication channel modeling. Based on this research, it is possible to clearly understand the IS design and the progress in the IS technology. Moreover, this paper presents a meaningful overview of the fundamentals, design principles (hardware and control), and selectivity of THz channels, which can be modified by the ISs.

### 1.2. Motivation and Contributions

Channel modeling is the basis for establishing reliable communication links. With the use of IS, wireless communication channels can be configured and controlled. Hence, the channel characteristics can be remodeled. The idea is to control the incident radiations on the surface, with constructive and destructive interference at the receiver’s location. ISs can reflect and phase shift the incident radiations to maximize the signal at the intended receiver while minimizing it to an unintended receiver.

In this context, this paper surveys the research works on IS-aided wireless communications, emphasizing channel modeling at the THz frequency band. In addition to reviewing the state-of-the-art and the IS principles, this paper outlines THz channel modeling and IS-aided THz channel models. This paper also includes an in-depth discussion and points out research issues related to THz channel modeling with the implementation of IS-aided wireless networks.

### 1.3. Organization of the Paper

This paper is organized as follows. Section 2 highlights some information about IS development, architecture, and main attributes. Section 3 summarizes THz channel modeling used to assess wireless communication system performance. Section 4 shows some IS-aided THz applications. Section 5 includes a brief comparison with other emerging technologies in 6G networks, a discussion about hardware and cost considerations, open issues, highlights, future trends, and research directions related to IS, THz, and both. Lastly, Section 6 closes the paper with its conclusion, main contributions, and content summary.

## 2. IS in Wireless Communication

This section provides an overview of the IS developments during the last decades, the IS architecture, and the main IS attributes that support SRE.

### 2.1. A Historical Perspective

Ancient people have already succeeded in controlling electromagnetic wave propagation. For example, the concepts of linear optics were stated more than 2400 years ago in China [64]. With the growth of studies and dissemination of knowledge over the globe, the beginning of the twenty-first century brought breakthrough discoveries on shaping electromagnetic waves as desired. Initially, simultaneous negative electric permittivity and magnetic permeability responses were achieved [65]. In addition, the negative index of refraction was verified experimentally [66]. Furthermore, they fabricated an electromagnetic invisible cloak [67]. Afterward, frequency-selective surface (FSS) were presented in [68], and the design for an absorbing metamaterial element was provided in [69]. Finally, metasurfaces and telecommunication systems were combined by introducing subwavelength thickness planar antenna arrays and generalizing the laws of reflection and refraction in [70].

In [71], the authors presented an experimental high-gain reconfigurable sectoral antenna that uses an active cylindrical FSS structure to obtain a directive radiation pattern. The structure was made of metallic discontinuous strips with positive-intrinsic-negative (PIN) diodes in their discontinuities and placed cylindrically around a dipole. Switching the diodes on the structure allowed a 360° radiation pattern sweep. In other words, the transmission and reflection coefficients of the FSS structure could be controlled by the state of the diodes.

An intelligent wall was proposed in [72], consisting of a wall equipped with an active FSS. These surfaces had PIN diodes fixed on the metal connection parts of each surface element that allowed the wall to behave as totally transparent or reflective in response to the incident radio waves. As reported in [72], the system performance of 80% was incrementally obtained using the proposed intelligent wall and a wireless cognitive network.

Kain et al. [73] used an electronically tunable metasurface as a spatial microwave modulator. To be placed on the wall, the metasurface was divided into 102 unit cells, defined as rectangular patches on a ground plane, spaced by half of the wavelength at the working frequency of 2.47 GHz. The resulting metasurface thickness was 1.5 mm. Each unit cell was a two states resonator that could reflect incident waves positively or negatively. The goal of the experiment conducted in a 36 $m^{3}$ office room was to maximize the transmission between two antennas, focusing the waves onto well-designed λ/2 isotropic focal spots or to minimize the electromagnetic field on an antenna placed at any location in the room.

Digital metamaterials, containing 30×30 particles, were proposed in [74]. Their metamaterial particles were two planar symmetrical metallic structures printed on the top surface of the F4B commercial dielectric board substrate and joined by a PIN diode. The total dimension of the particle was $0.172\times 0.172\times 0.057\lambda^{3}$ at the central frequency of 8.6 GHz. Applying a biased voltage of 3.3 V to the diodes, the particle behaved as a “1“ digitally coded material. When there was no biased voltage, it behaved as a “0” digitally coded material. The tests showed that the phase response difference between those states was exactly 180° at 8.6 GHz. The authors in [74] were able to alter the scattering pattern of normally incident beams on the digital metamaterial from being directly reflected to being reflected in two different directions, forming an angle of approximately 60°.

Huang et al. [75] proposed a method to model, analyze, and design graphene metasurface-based THz absorbers. They used the equivalent circuit model approach and designed a broadband and tunable absorber with an absorption over 70% fraction bandwidth. The results were verified with full-wave electromagnetic simulation. The model fitted THz sensing application since the peak absorption was maintained while tuning the peak frequencies.

Based on the modeling and design of tunable reflect-array elements in [76], Tan et al. [77] constructed smart reflect-arrays that were hung on the walls of a room to enhance the indoor spectrum sharing capacity by changing the phases of the reflected signals and changing the spatial distribution of the signal strength. The experiment showed that the interference was canceled to −73 dBm, and the signal-to-interference-plus-noise ratio (SINR) was increased to about 30 dB.

A programmable metasurface capable of modeling incident electromagnetic waves polarization, scattering, and focusing was proposed in [78]. Using a digital metasurface similar to the one in [74], the authors fabricated a prototype with 1600 metamaterial particles, obtaining an aperture size greater than 20 wavelengths. With coding matrices being used to control the PIN diode states of each particle, they obtained reconfigurable polarization conversion, scattering, planar focusing, beam steering, and beam forming.

In [79], the authors advanced the previously performed study in [80], using a 3D wireless modeling to simulate signal propagation in an indoor environment and an iterative stochastic optimization process to search for a 3D reflector shape to be put around an AP. The low-cost reflector, about US$30, provided a cheaper way to strengthen the AP signal in desired directions than using directional antennas. Their findings are 55.1% more throughput and a signal strength gain of 6 dB. Despite being a non-reconfigurable surface, the presented reflectors could optimize wireless coverage. The same authors of [74] proposed space-time coding digital metasurfaces in [81]. Based on the time modulation of the digital metamaterial particle’s reflection coefficient, they could control the metasurface scattering pattern and its harmonic power distribution. Switching coding sequences cyclically permitted redistributing the arriving radio wave and its odd harmonic frequencies in different directions.

LIS was proposed in [82] as an extension of mMIMO systems. Assuming that an entire surface is used as an intelligent receiving antenna array, the area is sufficiently large, and matched-filtering is employed, the authors derived the channel capacity for three different positioning scenarios of the terminals communicating with the LIS: along a line, a plane, and a cube. The conclusion was that the inter-user interference of two users at the LIS is close to a sinc-function. The inter-user interference is negligible as long as the distance between two users is larger than half of the wavelength. The equivalence between the LIS continuous surface model in [82] and the discrete dense grid of antennas model was assessed in [83] and corroborated in [84]. Indeed, the authors in [84] compared SINR theoretical and simulation results using a 128×128 LIS implementation with a λ/2 spacing at 5 GHz and observed that the mismatch could be reduced by decreasing the distance between the LIS points or augmenting the distance between the LIS and the user.

Metasurfaces incorporating networked hardware control elements were extensively detailed in [85]. They are called hypersurfaces tile-coat environmental objects and communicate with existing systems, such as localization services and cloud computing, to create the optimum wave scattering and combat path loss and multipath phenomena. The researchers elaborated on the first model to describe programmable wireless indoor environments, focusing on hardware, networking, and software components. They also evaluated the potential of programmable environments via a full 3D ray tracing in 2.4 and 60 GHz cases.

The smart wallpaper, called RFocus in [86], comprises 3200 antennas, each costing a few cents at scale, arranged on a 6 m^2^ surface. The surface operated passively, in the sense that it did not amplify the receiving signal but directed it only. Each element of the RFocus IS could operate in one of two possible states: “on” or “off”. The “on” state meant that the element would reflect the signal, and “off” that the signal would pass through it. The experiments in [86] revealed that the RFocus yielded an improvement of the median signal strength by 9.5× and the median channel capacity by 2×, and robustness to elements failure: for one-third of elements cutting out, the performance improvement drops 50%. The smart glass mentioned in [87] was a glass substrate covering a two-dimensional surface that had a large number of sub-wavelength unit elements placed in a periodic arrangement. The motion of the glass substrate enabled three modes of operation concerning the arriving radio waves: full penetration, partial reflection, and total reflection. Measurements obtained transmittance values in the range of −0.3 to −15 dB for frequencies varying from 26.8 to 27.3 GHz.

Recently proposed, intelligent omni-surface (IOS) represents an improvement for IRS because the latter leads to incomplete wireless coverage because it either reflects or does not the arriving signal. In [88], the authors studied an IOS-aided downlink cellular system, in which signals received through the IOS were transmitted and reflected concurrently. This behavior was obtained after embedding *N* diodes in the IRS metal patch element and connecting each one to the ground. They stated that $2^{N}$ phase shifts in total were possible for each element. Based on Monte Carlo simulation, the authors showed that by using IOS, the spectral efficiency could be doubled in comparison with the use of IRS.

Finally, the EU-funded Pathfinder project was started in 2021, aiming to set the theoretical and algorithmic bases of IS-empowered wireless 2.0 networks [89]. Carrying on the advancements in the area of electromagnetic metamaterials, the project’s long-term vision is to enable wireless channels to adapt to the operation of cellular networks. This will create a SRE in which radio waves circumvent physical obstacles; the signals will be combined at the receiver through the phase alignment of the IS elements, and sensors and IoT devices will “recycle” and backscatter the radio waves with IS.

### 2.2. IS Architecture

An IS is a flat structure made of specific materials, which are the base of the IS classification. Copper and graphene have been used. Researchers in wireless communications have focused on two main implementations: antenna-array-based and metasurface-based structures. The radio waves impinging on these electromagnetic material surfaces can be reconfigured to adjust the wireless propagation environment. By controlling the phase shifts and wavefront, the waves are reflected to propagate toward their desired directions. By making the wireless environment programmable and controllable, these surfaces monitor the propagation to improve the efficiency of the received signal.

The different types of IS found in the literature include active and passive surfaces, denominated according to the capacity to perform operations on the incident radio waves. In addition, ISs whose functions can or cannot be modified after manufacturing or deployment are denominated dynamic and static surfaces, respectively.

**Figure 1:**
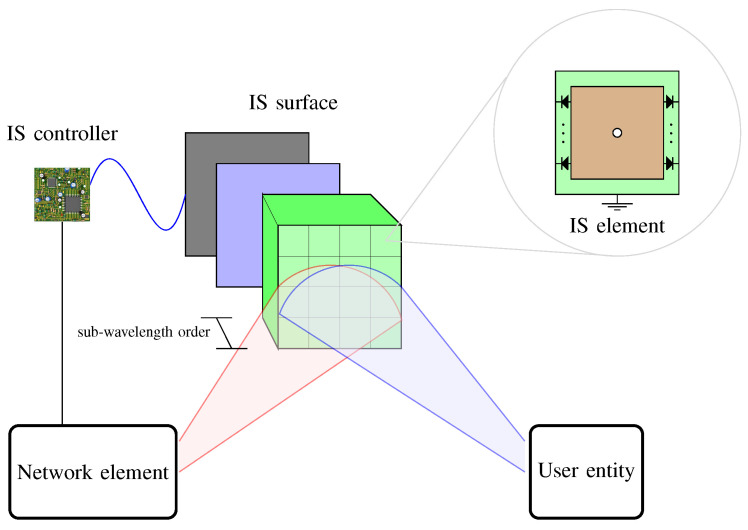
Example of IS architecture.

Figure 1 depicts the IS architecture shared among most technical papers in the literature. A top-down IS perspective view can be seen as a thin composite material sheet controlled electronically. Composite means that the planar structure is typically made of three layers. The reflecting elements constitute the outer layer, and the inner layer is a control circuit board responsible for adjusting the reflection amplitude and phase shift of the IS through feeding lines and via holes. The middle layer is a conductor between the outer and inner layers to avoid signal energy leakage. The IS controller is used for both programming the surface response and interacting with other network elements, such as base stations (BSs), APs, and user devices. Most prototypes have used field programmable gate array (FPGA) as the IS controller.

From a bottom-up perspective, the IS outer layer comprises multiple reflecting elements, or meta-atoms, of electric thickness. Electric thick means this reflecting element dimension is in the order of the sub-wavelength of the operating frequency. The meta-atom signal phase response is modified according to the electronic device states embedded in it. Devices used in practice include PIN diodes, field-effect transistors (FETs), micro-electromechanical systems (MEMSs) switches, and microcontrollers. Applying a biasing voltage to the feeding line energizes the reflecting element and the electronic devices through the via hole. The combination of the electronic device states defines the phase shift pattern. The meta-atom signal reflection amplitude can be controlled via a variable resistor in the element design. Varying the element resistance dissipates distinct portions of the arriving signal energy.

Finally, prototypes have combined iterative algorithms, integrated with the IS controller, to pursue the optimum configuration of each reflecting element, therefore achieving an efficient operation of the IS in accordance with the application. Such algorithms have also been used to alter the IS element states cyclically so that the reflected signal power is distributed harmonically.

### 2.3. IS Operation

Each IS surface element is considered the same size, i.e., they share the Δx width and Δy height. Also, they contain *N* PIN diodes assembled on dielectric substrates that connect the reflective element to the ground when the via hole is electrically charged. The IS element impedance is an association of all layers impedance [75].

**Figure 2:**
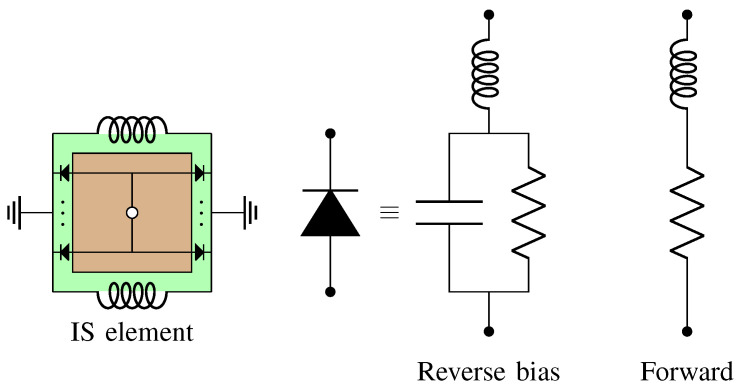
IS element and PIN diode state equivalent circuits.

As the IS controller applies a bias voltage to the feeding lines, the IS element is energized across the via hole, and the PIN diodes change their states between “on” and “off” accordingly. The point in incorporating PIN diodes is to adjust the IS element frequency response dynamically [72]. Figure 2 shows the equivalent circuit for the IS element and both PIN diode states. For a horizontally polarized incident wave, the ground bars around the IS element central patch act as inductors horizontally [68]. With the PIN diode insertion on the IS element, its reverse bias or forward equivalent circuits can be applied to estimate the transmission coefficient and 3 dB cut-off angular frequency of the IS element.

**Figure 3:**
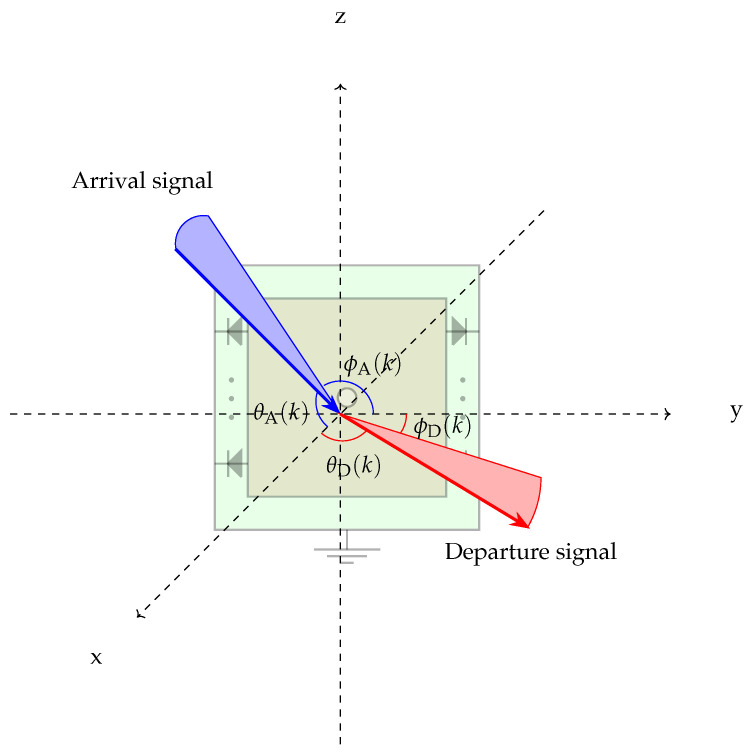
Angles of arrival and departure for a signal reaching the IS *k*-th element.

The combination of the *N* PIN diode states leads to distinct phase shifts for each IS element. As the signal arrives at the IS element, two other signals are formed: the transmissive and reflective signals. There are $2^{N}$ possible phase shifts within the angle of 2π rad [88]. Say, a signal arrives at the IS *k*-th element within the direction $\xi_{A}\left(k\right)=\left(\theta_{A}\left(k\right),\phi_{A}\left(k\right)\right)$, the corresponding reflected or transmitted signal is redirected by a phase shift ψ(k), obtained from the combination of the IS *k*-th element PIN diode states, such that the departure direction is $\xi_{D}\left(k\right)=\left(\theta_{D}\left(k\right),\phi_{D}\left(k\right)\right)$. The IS element phase shift determines the transmissive and reflective signal directions concurrently. Figure 3 illustrates the angles of arrival and departure for a signal reaching an IS *k*-th element. The phase shift imposed by every IS element accounts for the IS surface phase shift. If there are *m* IS elements, the phase shifts of the IS surface can be written as the vector $\phi_{\mathrm{IS}}=\left[\phi_{D}\left(1\right),\cdots ,\phi_{D}\left(m\right)\right]$.

The influence of the IS *k*-th element over the upcoming signal was experimentally obtained as a function of the arrival direction, departure direction, and the phase shift as [88]

(1) $$g_{k}\left(\xi_{A}\left(k\right),\right.\left.\xi_{D}\left(k\right),\psi \left(k\right)\right)=\sqrt{G_{k}K_{A}\left(k\right)K_{D}\left(k\right)\Delta x\Delta y{\left|\gamma_{k}\right|}^{2}}e^{-j\psi (k)},$$

in which $G_{k}$ is the antenna gain of the IS *k*-th element, $\gamma_{k}$ is the power ratio of the departure signal to the arrival signal, and $K_{A}\left(k\right)$ and $K_{D}\left(k\right)$ are the normalized power radiation pattern of the arrival signal and departure signal, respectively. This influence is a complex number that forces a gain and phase shift to the departure signal.

## 3. THz Channel Modeling

The THz frequency bands beyond 100 GHz and below 10 THz comprise a still largely unexplored part of the electromagnetic spectrum. They can adequately meet the demands of the 6G networks due to their multiple gigahertz of bandwidth [4]. This multi-gigahertz bandwidth opportunity can increase the number of users, provide signal location and detection, and satisfy some of the requirements of the next generation of wireless networks. As a result, THz communication will allow higher data rates with lower latencies [90]. Despite their large bandwidth, THz communication suffers from strong atmospheric attenuation, molecular absorption, and severe path loss, limiting its propagation [91]. These drawbacks make the implementation of a THz communication system very challenging.

It can be said that part of the most substantial restrictions regarding the design of a communication system is imposed by the channel through which the signal is transmitted [92]. Thus, the increase in frequency requires an analysis of the best-explored propagation routes. Furthermore, the increasing demand for efficient communication services has led devices to operate in a system composed of different channel environments [93]. In this context, the adequate modeling of the wireless channel is essential, mainly in the THz band.

The channel models in THz can be classified as statistical, deterministic, and hybrid, depending on the number of antennas implemented [94]. This study exploits the deterministic and statistical models of single-input single-output (SISO) and MIMO systems.

### 3.1. Deterministic Channel Model for THz Band

Generally, the two deterministic models found in the literature are the ray-tracing (RT) and the finite-difference time domain (FDTD) models [95,96,97,98]. These deterministic channel models are based on the theory of electromagnetic waves. However, their properties depend on the materials, propagation medium, and spatial positions of transmitter and receiver antennas. In this subsection, the RT is explored firstly and the FDTD secondly.

**Figure 4:**
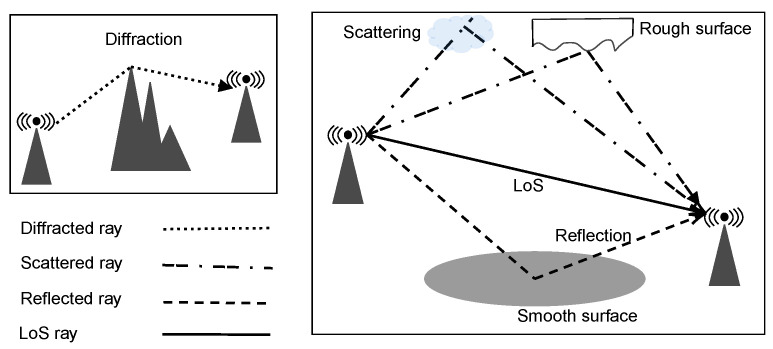
The ray-tracing method for deterministic channel modeling.

Wireless communication channels are well-known to be subjected to the multipath phenomenon [99,100]. This phenomenon occurs when the transmitted radio signal finds multiple paths between the transmitter and the receiver. However, its occurrence is connected to the propagation environment and the obstacles between the transmitter and receiver. Figure 4 shows from a pictorial perspective the main propagation phenomena.

As mentioned before, the RT technique analyzes the propagation of electromagnetic waves for each of these geometric paths. The absorption by water vapor molecules is one of the main factors that affect the propagation of waves in the THz band [101]. In addition to introducing thermal noise, absorption causes an attenuation of the signal sent to the receiver. As occurs in the most explored bands, also in the THz band, the propagation in free space must consider the propagation loss. Complete ray tracing models were developed for 0.06–10 THz [95] and 0.3 THz [96] frequency bands, respectively.

According to [95], the THz channel band can be modeled as the superposition of line of sight (LoS), reflected, scattered, and diffracted waves attenuation related to many individual sub-bands. Furthermore, the propagation delay experienced by each wave is taken into account as per the path it traverses.

The LoS channel transfer function can be represented by

(2) $$H_{\mathrm{LoS}}\left(f\right)=H_{\mathrm{Spr}}\left(f\right)H_{\mathrm{Abs}}\left(f\right)e^{-j2\pi f\tau_{\mathrm{LoS}}},$$

where $H_{\mathrm{Spr}}\left(f\right)$ is a spreading loss function, $H_{\mathrm{Abs}}\left(f\right)$ is a molecular absorption loss function, $\tau_{\mathrm{LoS}}$ is the delay and *f* is the observed frequency.

The channel transfer function of the reflected rays can be written according to

(3) $$H_{\mathrm{Ref}}\left(f\right)=\left(\frac{c}{4\pi f\left(r_{1}+r_{2}\right)}\right)e^{-j2\pi f\tau_{\mathrm{Ref}}-\frac{1}{2}k\left(f\right)\left(r_{1}+r_{2}\right)}R\left(f\right),$$

in which *c* is the speed of light, $\tau_{\mathrm{Ref}}$ is the time-of-arrival of the reflected ray, $r_{1}$ is the distance between the transmitter and the reflector, $r_{2}$ the distance between the reflector and the receiver, R(f) is the reflection coefficient, and k(f) is the absorption loss.

The channel transfer function of the scattered ray propagation is

(4) $$H_{\mathrm{Sca}}\left(f\right)=\left(\frac{c}{4\pi f\left(s_{1}+s_{2}\right)}\right)e^{-j2\pi f\tau_{\mathrm{Sca}}-\frac{1}{2}k\left(f\right)\left(s_{1}+s_{2}\right)}S\left(f\right),$$

where $\tau_{\mathrm{Sca}}$ is the time-of-arrival of the scattered ray, $s_{1}$ is the distance between the transmitter and the scattering point, $s_{2}$ is the distance between the scattering point and the receiver, and S(f) is the scattering coefficient.

Finally, the transfer function of the channel in the diffraction ray can be written as

(5) $$H_{\mathrm{Dif}}\left(f\right)=\left(\frac{c}{4\pi f\left(d_{1}+d_{2}\right)}\right)e^{-j2\pi f\tau_{\mathrm{Dif}}-\frac{1}{2}k\left(f\right)\left(d_{1}+d_{2}\right)}L\left(f\right),$$

in which $\tau_{\mathrm{Dif}}$ is the time of arrival of the diffracted ray, $d_{1}$ is the distance between the transmitter and the diffracting point, $d_{2}$ is the distance between the diffracting point and the receiver, and L(f) is the diffracting coefficient.

Those different signal components are added to the receiver in the RT technique. Another way of characterizing THz waves is through Maxwell’s equations [102]. They predict the propagation of electromagnetic waves through magnetic and electric fields and, in contrast to the RT technique, Maxwell’s equations incorporate antennas and other structures.

Various authors have explored Maxwell’s equations to model THz electromagnetic wave propagation. For example, in [97,98], numerical analysis techniques that approximate the magnetic and electric fields in temporal and spatial domains for THz waves are proposed. Those techniques, denoted FDTD, can resolve small and complex scatterers and rough surfaces in the THz band. However, this approach relies on computers’ memory capacity, as they provide numerical solutions to the electric and magnetic field equations used to model the THz electromagnetic wave propagation.

### 3.2. Modeling of Statistical Channels

The previously presented deterministic models provide accurate channel modeling results at the cost of high computational complexity. As a result, elaborating deterministic channel models can be far too time-consuming [103]. In addition, geometric knowledge of the propagation environment is required for those models. Statistical propagation models were proposed and designed to overcome those limitations, and numerous models are found in the literature. Some common models are the Rayleigh [104], Nakagami [105], and Rice [106] models. However, in general, they are used to represent narrowband fading and do not provide a complete analysis of the physical phenomena that occur in the THz band.

For fast channel realizations, a statistical impulse response model for the range 275–325 GHz was presented in [107]. The proposed approach includes fully polarimetric path amplitudes, amplitude-frequency dispersion, phases, direction of departure (DoD), direction of arrival (DoA), time of arrival (ToA), propagation phenomena, and antenna characteristics, and is suitable for multi-antenna systems. The authors conducted RT simulations for 3201 frequency points and then compared data histograms with analytical distribution models. Based on the results obtained by the authors, they provided parameter values that can be used for a universal stochastic spatiotemporal model to generate ultra-broadband THz indoor radio channel realizations.

In addition, [108] proposes a RT-statistical hybrid channel model for THz indoor communication in the range 130–143 GHz. The measurement campaigns were made in an indoor environment, and the calculations considered the same environment. The approach provided insights into THz waves propagation including LoS path loss, power-delay-angular profiles, delay and power spreads, and correlations among THz multipath characteristics such as antennas separation and the number of clusters. The hybrid model showed better results due to its adaptive capacity within the measurements in different positions between the transmitter and the receiver.

## 4. IS-Aided THz Channels

IS has been considered a promising technology and an indispensable part of THz communication systems. As channels in the THz band are highly frequency-selective, IS applications in these frequencies can solve the limitations on propagation distance and coverage, which are easily affected in THz frequencies due to very high propagation attenuation and molecular absorption [101]. In addition, it can provide an additional propagation path where NLoS link exists and, consequently, assist the communication where the LoS path is blocked by obstacles [14]. Therefore, the IS ability to create alternative signal paths makes IS applications essential in THz wireless communication, where the environment introduces several challenges, as described in the preceding sections.

In addition to creating additional links between the transmitter and receiver, the ISs also use wireless mm-wave intercell communication for tunability. The IS architecture is based on FPGAs, which are responsible for enabling the control and intelligence of IS. To transfer information among unit cells, wireless signals not leaked outside the IS, and no interference with the network, frequency and time domain and path propagation analysis have been performed [109,110,111]. The thorough analysis of the authors has provided that wireless communication to control the IS is an optimal choice over wired communication for large or dense IS employments. Besides that, out of three options of communication channels: the chip layer, a dedicated parallel-plate waveguide, and inside the IS dielectric; the first option yields the most considerable path loss (40–50 dB) while the third option shows the smallest one (20–40 dB). However, the IS design must consider its dispersion factor while enabling its work at the targeted frequency.

**Figure 5:**
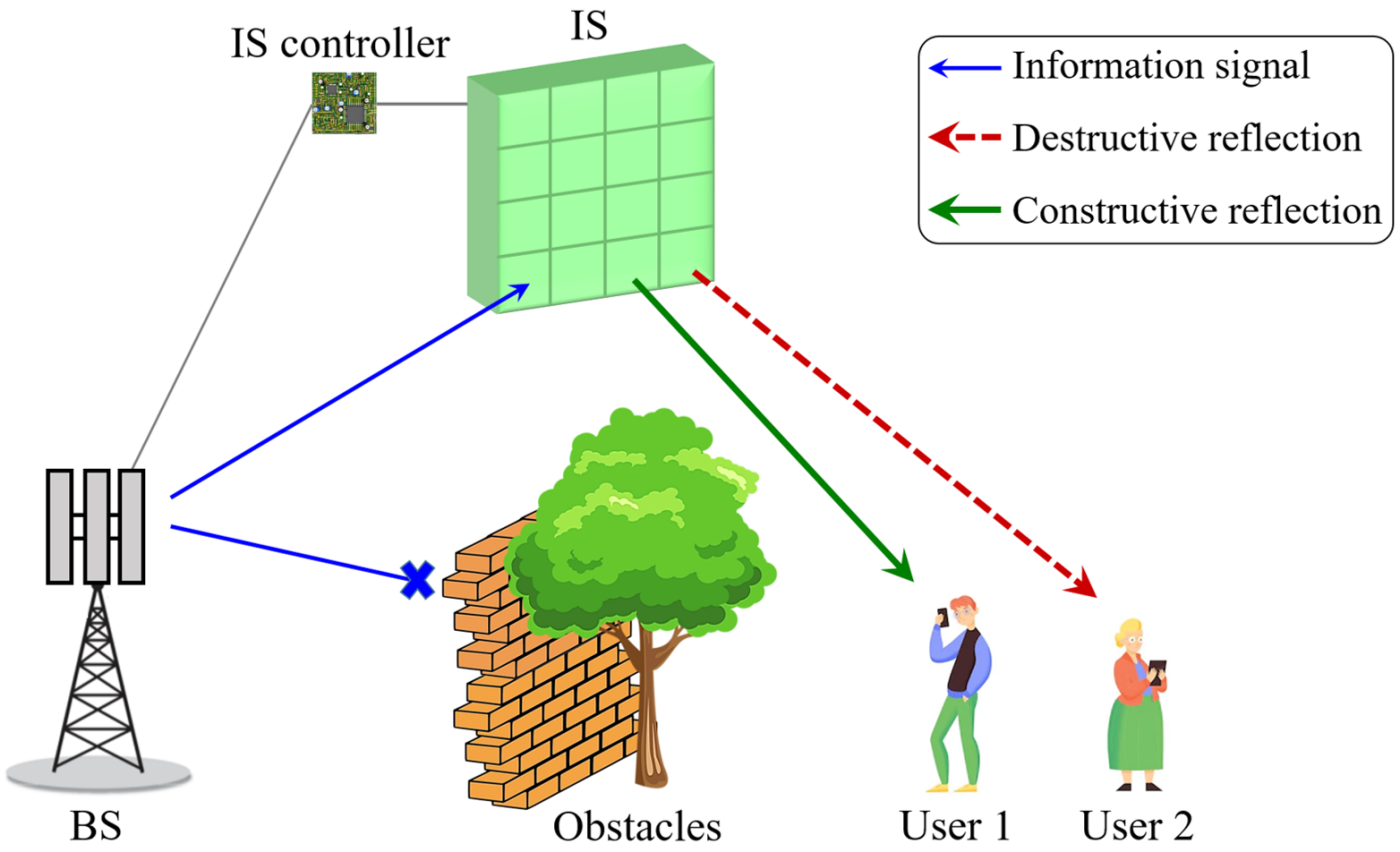
Example of communication with IS in THz band.

Moving from the perspective of the IS controlling channel to the one used to enable communication, Figure 5 shows a typical application scheme of an IS for THz communication. As shown, User 1 desires to connect to the BS; thus, the system requires a LoS link between User 1 and the BS. Nonetheless, the wireless link is blocked by obstacles. In a traditional approach, the User 1 would try to find another BS with a weaker signal due to the longer distance from User 1. Also, high path loss bounds THz waves to a short propagation range [112]. However, there is an IS with a total $m=m_{x}m_{y}$ elements, where $m_{x}$ accounts for the number of elements in each row and $m_{y}$ accounts for the number of elements in each column. Due to the IS installation position, the signals from the BS can be transmitted to User 1 by reflection from the IS. The changes in directions of the reflected beam are controllable, according to the generalized Snell’s law, which is derived from Fermat’s principle to interpret the anomalous reflection and refraction phenomena [113]. This means that when the IS elements alter the phase and magnitude of the incident waves, occurs constructive or destructive interference of these incident waves with the reflected waves from IS in desired directions. Each element introduces a phase shift, ideally given by the generalized Snell’s law to create a single beam. Still, the phase shift must be explicitly optimized since a superposition of multiple beams should be created.

The information sent to the desired user in Figure 5 must be received only by User 1. Therefore, the information-bearing signal and the reflected signal constructively interfere, and consequently, a constructive reflection is sent toward the desired user (User 1). There is also an interfering user (User 2) that must not participate in the communication between User 1 and the BS. Therefore, destructive interference occurs between the waves that are incident on IS and those that are reflected in the direction of User 2. In this case, there is no communication path between User 2 and BS, so that information remains secure with the desired user. As ISs can reduce the propagation loss with intelligent reflections in order to enhance the end-to-end system performance [28], channel modeling strategies for IS-aided communications systems are fundamental to provide detailed channel characteristics.

In that context, [53,114] evaluated the system performance when the LoS path from the transmitter to the receiver in a downlink THz indoor wireless communication is blocked. The system performance under the phenomenon of the receiver antenna beam misalignment was evaluated in terms of average SNR, ergodic capacity, and OP in [53]. The phase shift that each IS element must impose to steer the radiated beam towards the receiver in terms of the channel attenuation and its cumulative distribution function are presented in [114]. The study in [53] showed that receiver beam misalignment deteriorates the system performance, but increasing the IS size improves overall system performance. The authors in [114] showed the importance of considering the molecular absorption loss for IS-aided THz channel estimation and that there is a minimum transmitter power that can guarantee a high coverage probability.

The system was modeled similarly to Figure 3 with the IS placed on the origin of *x*-*y* plane, the dimensions of the IS elements, denoted by $d_{x}$ and $d_{y}$, were in the range $\left[\lambda /10,\lambda /2\right]$ [115], and the transmitter and receiver located at distances $d_{1}$ and $d_{2}$ from the IS center, respectively. The arrival and departure angles of the signals from the transmitter and to the receiver in relation to the IS center, respectively, were then determined. Based on these assumptions, the resulting expression for the channel gain |h| at the receiver is given as [53,114]

(6) $$\left|h\right|=\frac{m_{x}m_{y}\lambda \left|R\right|}{8d_{1}d_{2}\sqrt{\pi^{3}}}\sqrt{\tau \left(f,d_{1}+d_{2}\right)}\sqrt{d_{x}d_{y}F_{t}F_{r}G_{t}G_{r}G_{\mathrm{IS}}\left(\theta_{r},\phi_{r}+\epsilon_{r}\right)},$$

in which |R|, τ(·,·), $F_{t}$, $F_{r}$, $G_{t}$, $G_{r}$, $G_{\mathrm{IS}}\left(\cdot ,\cdot \right)$, $\theta_{r}$, $\phi_{r}$, and $\epsilon_{r}$ stand for the amplitude of the programmable reflection coefficient of each IS unit cell, transmittance of the absorbing atmospheric medium [116], transmitter normalized power radiation pattern, receiver normalized power radiation pattern, transmitter antenna gain, receiver antenna gain, an IS unit cell gain, receiver antenna bandwidth, angle of boresight direction, and receiver beam steering error angle, respectively. The IS unit cell gain $G_{\mathrm{IS}}\left(\cdot ,\cdot \right)$ is calculated as [53]

(7) $$G_{\mathrm{IS}}\left(\theta_{r},\phi_{r}+\epsilon_{r}\right)=\frac{2\pi q_{r}}{\theta_{r}\left(q_{r}+1\right)}U\left(\frac{\theta_{r}}{2}-\left|\phi_{r}+\epsilon_{r}\right|\right)+\frac{2\pi }{\left(2\pi -\theta_{r}\right)\left(q_{r}+1\right)}U\left(|\phi_{r}+\epsilon_{r}|-\frac{\theta_{r}}{2}\right),$$

in which U(·), $q_{r}=\frac{2\pi }{b_{r}\left(2\pi -\theta_{r}\right)}$, and $b_{r}$ represent the unit step function, forward-to-backward power ratio, and an antenna specific constant.

Physically, it can be seen from the previous expressions that each IS unit cell impacts the channel gain at the receiver by means of their normalized received power ratio, aperture, phase shift, and reflection coefficient (see Appendix A in [114]). THz channels impairments are mainly represented by the transmittance of the absorbing atmospheric medium. Stochastically, the channel gain at the receiver is interpreted as the summation of the order of the IS size of products of channel coefficients between the transmitter and the IS and the IS and the receiver [28]. Mathematically,

(8) $$\left|h\right|=\sum_{i=1}^{N}h_{i}^{\mathrm{Tx}}g_{i}h_{i}^{\mathrm{Rx}},$$

in which *N*, $h_{i}^{\mathrm{Tx}}$, $g_{i}$, $h_{i}^{\mathrm{Rx}}$ are the number of IS elements, transmitter to IS channel coefficient, *i*-th IS element response, and IS to receiver channel coefficient. All components on the right side of (8) are composed of amplitude and phase, and researchers consider the IS controller knows the channel, producing phase shifts that cancel out the phase response of the channels [16]. In addition, IS are assumed to work passively and have unitary amplitude. The different probabilistic channel models used to investigate IS-aided THz channels have been mixture Gamma [117], Rayleigh [118,119], Rice [120,121], Nakagami [120,122,123,124], and shadowed κ-μ [125] models.

Due to the intelligent reflections, an IS-aided communication has dynamic control capability over the wireless propagation channel phenomenon. This IS capacity to change propagation characteristics of wireless channels promotes the development of the THz communication systems to meet the ever-increasing user demand for higher data rates. The benefits of the IS applications in the next-generation wireless technologies depend on the development of an accurate channel model, which is necessary for designing and deploying future wireless communication systems. However, only the study in [107] exploits this development in THz band. The authors proposed the first statistical model for channels in THz, where extensive ray tracing simulations were performed to obtain the statistical parameters of the channel. Technical challenges arise when an IS channel modeling is incorporated in a system project, such as mobility of users or ISs and optimization of the phase shift of the IS. From this perspective, the THz propagation channel modeling involving modification of the wireless propagation environment through the inclusion of IS has attracted the attention of researchers in recent years.

To enable the efficient transmission of THz waves, which suffer because of high path loss, the IS operation in THz wireless communication links has been analyzed for the proposed path loss models. For instance, the study in [31] employs electromagnetic theory to deduce the expression that returns the phase shifting of each IS reflection unit. Also, Refs [32,41] proposed a path loss model at THz by using physical optics from electromagnetic theory to elaborate a path loss expression. The work in [41] assumes far-field path loss with a plane-wave approximation for the THz channel model, and [32] provides a near-field channel model for path loss calculation, taking into account the spherical wavefront of the radiated waves. To study realistic path loss models for IS-aided THz wireless networks, it is necessary to find the IS link budget potential as well as their optimal placement in space.

The studies employing spherical wavefront for the THz waves show that when the source emits an electromagnetic wave reflected to the receiver through the IS, the reflected signal will present a narrow beam. As THz waves encounter high propagation losses and short wavelengths, many reflecting elements are expected to compensate for the severe impairments. Due to their beam width, a THz IS operates in its Fresnel zone, resulting in a radiating near-field that covers several meters. Near-field conditions bring the possibility of focusing the signal towards a specific location in space instead of a specific direction, as in the conventional far-field assumption. In such setups, ISs are expected to communicate with multiple users, possibly operating in the near-field condition. The authors in [51] used the spherical wave channel model to analytically determine the expression for the path loss and beam pattern of a holographic IS in the radiating near-field, i.e., in the Fresnel zone. They proposed a holographic control of THz IS with the capability of generating multiple predefined shaped beams in several directions to determine the field scattered. The holographic control of electromagnetic waves can create versatile radiated beams, eliminating the need for hardware with complex circuits.

Another way to combat the THz drawbacks on the propagated waves is implementing the mMIMO antenna systems. The application of holographic ISs in the THz mMIMO systems is proposed in [52], where the channel is modeled considering the IS-aided THz mMIMO system over a frequency-selective fading channel. Based on the Fourier transform, the authors introduce an angular domain beamforming framework and obtain closed-form expressions for the beamforming design in two specific cases. In order to reduce the uplink pilot-overhead, a compressed sensing-based channel estimation algorithm is proposed, where the sparsity of the THz MIMO channel is exploited in both the angular domain and the delay domain. They consider the system operating in time division duplex (TDD) mode and consequently assume channel reciprocity between uplink and downlink. This work shows the effectiveness of the holographic IS on THz MIMO channels supporting THz mMIMO systems.

In [50], the small-scale amplitude fading of THz signals is modeled with the help of previous measurement data by using the fluctuating two-ray (FTR) distributions [126]. FTR fading model can fit the signal amplitude fading of THz communication in the IS-aided channel modeling. A swarm intelligence algorithm obtains the phase shifts under discrete phase-shift constraints. Essential performance metrics were obtained based on real THz signal measurements, essential performance metrics were obtained, including the OP and ergodic capacity. The authors derived expressions of OP for a IS-aided THz communications system. They obtained an expression for ideal radio frequency chains for the ergodic capacity.

A THz IS can establish favorable wireless channel responses by controlling the multipath and the diversity of the wireless propagation environment through its elements. By exploiting UAV, real-time adjustments can effectively mitigate the multipath and Doppler effects caused by the movement. The authors in [54] jointly optimized the UAVs trajectory, the continuous phase shift of IS, power control, and the allocation of THz sub-bands. They determined the UAV trajectory and IS phase-shift by closed-form expressions using a successive convex approximation (SCA) algorithm [127]. The mobility of devices challenges the THz link reliability and THz network performance. By directing beams toward the receiver to enable THz communication links, ISs can solve those issues and ensure mobile THz communication. Thus, the feasibility of mobile THz wireless communication systems of the THz links has been one of the pressing as well as challenging research targets. Considering satellite networks, Ref. [128] showed that the integration of IS and low Earth orbit satellites can create a sophisticated architecture to operate at 350 GHz that can improve the SNR with the increasing of IS elements. In [128], the authors concluded that the error rate performance is proportional to the number of IS elements and satellites in the form of $P_{e}\propto N^{2M}$, where $P_{e}$, *N*, and *M* are the bit error rate, number of IS elements, and the number of satellites, respectively.

Table 2 compares the related articles and explores some aspects of the THz communication channels. The topics cover channel modeling characteristics at THz band, including performance evaluation models, system setup for performing channel design objectives, and channel optimization techniques.

## 5. Discussions and Research Directions

### 5.1. Comparative Analysis: Emerging Technologies

Besides the IS techniques mentioned throughout this survey to improve THz communications, other emerging technologies can be used for the same purpose. An emerging antenna technology with great potential to impact the development of 6G wireless networks is the fluid antenna system (FAS) [134]. It offers additional diversity and multiplexing benefits by exploiting dynamic radiating structures. As an example of a possible implementation, the antenna radiating elements can be installed in a tube-like structure within which a fluid can move freely among preset positions known as ports. Thus, the FAS structure changes its position and shape according to characteristics such as gain, radiation pattern, and operating frequency bandwidth [135]. By exploring FAS flexibility in its shape and positions, the authors in [136] analyzed the OP to evaluate the FAS performance to combat high propagation path loss of THz communications. The research on FAS is still in its early stages, but it is advancing quickly. Some FAS limitations have already been pointed out and need to be overcome, such as the correlation model [137], how to achieve fast switching among the ports, and the optimal algorithm for the selection of the ports [138].

Similar to FAS, liquid crystal (LC) has been explored for THz communication applications due to their potential as a tunable dielectric material. To overcome the challenge of high propagation loss, LC is modeled to construct antenna structures that radiate efficiently, obtaining large tunability and fast switching [139]. As they can direct THz waves dynamically, LC antennas are an interesting solution for THz communications. The work in [140] proposed a programmable metasurface based on LC, where a phase change could be flexible by switching between 0 and 1 states of each element.

Besides FAS and LC antennas, and different from conventional mMIMO systems, CF mMIMO is a distributed architecture that has also been introduced as a promising technology for enabling future 6G networks [141]. This network architecture comprises numerous AP in a coverage area to serve users close to the APs. In [131], the IS-aided CF mMIMO system is based on the CF architecture with an additional IS layer between the AP and its users. The authors analyzed the network performance over spatially correlated fading and the blocking probability of the direct AP-user links.

Finally, machine learning or AI must be considered as another fundamental 6G enabler, where simple and complex algorithms can be trained to provide intelligent solutions and efficient multiple access resource allocation under the premise of maintaining the coexistence between THz and lower frequency bands [142].

### 5.2. Hardware and Cost Considerations

To meet the hardware requirements for THz communication, it is imperative to have specialized devices capable of operating within the corresponding frequency band. These devices must demonstrate the ability to transmit and receive signals at high speeds, with a modulation bandwidth of at least 10 GHz [143]. In addition, they must possess an optimum combination of transmit power, antenna gain, and receiver sensitivity, meeting the link budget requirements according to the propagation conditions and the target communication distance [143]. Energy efficiency, low power consumption, and reconfigurability are critical in developing smart, dynamic, and sustainable THz communication and sensor systems, especially in future 6G networks and beyond [144]. These features raise the cost compared to lower-band hardware because of the need for specialized technology to operate in the THz frequency band [143]. Additionally, experimental THz platforms are expensive to build, limiting the ability to evaluate the performance of large THz networks [143]. Although simulation platforms represent a cheaper alternative, they are often based on abstractions or mathematical models of device components and wireless channels. Hardware-in-the-loop emulation platforms may offer an intermediate alternative to more accurately capture the actual behavior of THz devices and channels. However, building and maintaining those platforms can also carry significant costs.

Hardware operating at THz band can also leverage IS to enable the transference of data on a chip-scale architecture with low latency and small power absorption for wireless networks on chips (WiNoCs) [145]. WiNoCs are used as communication backbones for integration in multi-core system-on-chip (SoC), which are appointed to provide an integrated solution to challenging design problems in the telecommunications, multimedia, and consumer electronic domains [146,147]. Other investigated solutions to overcome the power and latency constraints of WiNoC are express virtual channels [148], photonic interconnections [149,150], and radio frequency communication [147,151]. In general, wireless and wired connections have been studied to satisfy the performance requirements. As for WiNoC inter-chip communication occurring in the THz range, the signals can suffer severe attenuation. As previously stated, IS are employed to shape channel impulse response at mmWave and THz frequencies [133,152]. With the detailed design of the on-chip IS, considering the impact of the thickness of the silicon layer over the signal attenuation and inter-symbol interference, the results have shown that IS can provide data transmission rates in on-off keying modulation with equalized wireless on-chip channels and double the permissible modulation speed for a given desired bit error rate value at a given noise level.

### 5.3. Practical Application Examples

To assess the practicality of IS-assisted THz communications research and address the challenges associated with erratic THz signal interference caused by mobile and human blockage, the authors in [129] conducted a case study using a 3D mapping environment. The study was developed in a multi-receiver propagation environment incorporating the implementation of multi-dimensional and distributed IS. The proposal promised to significantly increase the SNR, improve the overall success rate and energy efficiency, and maintain optimal quality of service (QoS) with adequate latency. These objectives were achieved by implementing a distributed IS framework algorithm designed to operate collaboratively. The authors considered practical factors such as complexity in IS system design, IS hardware constraints due to limited volume, high value of state-of-the-art THz hardware, accuracy of location information, and feasibility of deployment in a realistic environment. Very recently, driven by the advantages of the distributed IS scheme, the authors of [130] have proposed the double-layer true-time delay with sparse radio frequency chain antenna structures for IS-assisted communication in THz, aiming to improve the system performance.

Some proof-of-concept prototypes of IS in wireless communication environments, specifically in the 5.8 GHz and sub-6 GHz bands, have been presented, where their potential benefits are evaluated under realistic conditions at these frequencies [153,154]. The evaluations revealed significant SNR improvements and coverage in blocked or hidden areas, enhancing higher-quality communications. Although the authors focused on the 5.8 GHz and sub-6 GHz bands, they ensured the adaptability of the prototype, especially in the design of simplified unit cells, as reported in [153]. This adaptability would allow for the practical fabrication of ISs at mmWave and THz frequencies.

Last but not least, in various high-speed and low-latency applications that are emerging as candidates for the future communication networks, such as UAVs, backscatter communication, mmWave utilization, and MIMO systems, ISs play a crucial role in boosting coverage, capacity, efficiency, and signal quality [155]. This is achieved by bypassing obstacles and reducing interference through skillful manipulation of radio signals. Additionally, in the context of non-orthogonal multiple access (NOMA) and simultaneous wireless information and power transfer (SWIPT), ISs contribute significantly by improving spectral efficiency, optimizing power transfer, and enhancing signal quality through their ability to manipulate radio signals [155].

### 5.4. Research Directions

To meet the 6G implementation requirements, IS will play an essential role in being able to decrease the signal attenuation THz and enable SRE [156,157]. Although significant progress has been made in several studies in THz signal research, some focus specifically on GHz technology [158,159]. On the other hand, it is crucial to consider that performance evaluations can be produced more accurately by considering THz signals instead of GHz signals. According to study [91], the THz band is subject to high attenuation and has a more limited propagation range than the GHz band. Critical reports can contain project cost analysis, device design, computational load, and IS sizing. In addition, research has modeled the influence of different IS elements on the transmitter and IS and IS and receiver links as independent, while in practice, they may be correlated. This relationship could exist depending on the distance between the IS elements. In addition to dealing with such a complex situation, researchers may find new obstacles to plan IS to maintain or exceed the performance metrics reported in previous works.

Knowledge of the channel makes it possible for transmitters and receivers to use this information to decide when to transmit in order to increase the probability of decoding at the receiver [160,161]. In addition, correctly modeling the propagation environment by considering both small-scale and large-scale fading makes the system more realistic [162]. Therefore, channel models are crucial for systems requiring high QoS. Comparisons between deterministic and statistical models for channel modeling THz were studied in Section 3. However, despite advances in the various proposals for propagation models at THz, the properties of materials in the high THz band remain unknown [163]. In addition, the frequency selectivity phenomenon in the THz channel deserves special attention due to the distortion in the pulse shape [164]. The statistical model must consider this phenomenon, as it may lengthen the pulse duration [165]. In addition, the dependence between DoA and DoD remains to be studied once it can be correlated [166]. Also, the clustering phenomenon at THz frequencies must be treated carefully, given its dependence on reflection and scattering properties.

To study the performance of IS in a more realistic scenario, with the complexity and amount of environmental information of THz channels, accurate modeling for both the IS element and the IS channel, is needed [156,162,165]. For channel modeling, when an IS is included, extensive work has been conducted to develop a sufficiently accurate model with acceptable complexity. Additionally, real-time optimization of IS phase-matrix is necessary to enhance the overall system performance [167]. With a massive number of reflecting unit elements to compensate for the severe propagation losses, existing methods of THz communication channel modeling, offset by intelligent signal processing algorithms and wireless connectivity techniques, require high-performance computing [168]. The idea is to apply machine learning tools and AI methods with affordable computational complexity to facilitate the reconfiguration of the IS phase matrix [169]. A THz wireless communication system based on the IS can be a secure communication method because it needs to know the mapping relationship between the received signal and the IS to reflect the transmitted information properly.

The chip-scale communication also deserves special attention regarding further investigation of IS application. As pointed out in [133,152], it is possible to conduct a more in-depth analysis of intricate scenarios, such as the ones involving one transmitter and multiple receivers. The analysis of WiNoC can also be improved by incorporating different wave processing in scattering enclosures like matrix multiplication, signal differentiation, and reservoir computing [170,171,172]. IS-aided WiNoC can be additionally integrated into machine learning models to enable the collaboration of multi-core chips [173,174]. The communication channels, including the IS-to-WiNoC, can also be better modeled to incorporate objects, such as racks, blades, chassis, and data centers [175,176]. Finally, other mmWave and THz ranges are worthy of exploration because they can enrich the present results of investigating IS-supported WiNoC using THz signals for communication, as there are still a few published results [133,152].

## 6. Conclusions

IS is a new technology that is emerging nowadays. However, in recent years, various works have pointed to it as an opportunity to improve the wireless communication system for the next generations. This is due to the improvements obtained mainly for THz communication. This paper reviewed the impact of the IS on THz communication modeling. Furthermore, it summarized the architecture of the IS, THz propagation models, and IS-assisted THz propagation models. Finally, it discussed the challenges that need further exploration and examination for future work. Although there is little variety of work on this topic, a great effort by researchers has been made due to the great potential, more importantly in THz communication. It highlights the opportunities that must still be addressed before next-generation network technology employs IS and achieves higher KPIs than the 5G.

## Figures and Tables

**Table 1:** Summary of surveys, magazines, and review papers related to IS in THz communication channel modeling and the classification according to discussion level of the approach taken on both the communication channel and IS.

Reference	Summary and Focus	Approach Taken on the Subject: References, Details and Future Directions	
		Channel Discussion	IS Discussion
[1]	Comparison between 5G and 6G, use cases for 6G enabling techniques, and beyond 6G technologies.	Deep	Intermediary
[2]	A survey of 6G wireless channel with future research challenges and IRS models.	Deep	Low
[3]	Application scenarios for THz RIS, enabling technologies and open issues.	Intermediary	Deep
[4]	THz technical challenges and performance issue solutions.	Intermediary	None
[14]	Overview of RIS technology and the key challenges in implementing a RIS-aided hybrid network.	Specific	Specific
[28]	A survey of RIS-empowered wireless networks.	Deep	Deep
[29]	Performance evaluation of RIS assisted D-band wireless communication.	Specific	Specific
[30]	TE-GD scheme is developed during the iterative process by a dynamic update of the step size in IRS assisted THz MIMO system.	Specific	Specific
[31]	Analytical path loss model for THz RIS.	Specific	Specific
[32]	THz IRS in MIMO systems operating in the near-field for beamfocusing and power gain MIMO.	Specific	Specific
[33]	Channel estimation and transmission solutions for mMIMO-assisted THz system.	Specific	Specific
[34]	Channel acquisition for the IRS enabled THz mMIMO system.	Specific	Specific
[35]	CSI and optimal data rate for IRS THz MIMO Systems.	Specific	Specific
[36]	IRS with beamforming optimization based on statistical CSI knowledge.	Specific	Specific
[37]	Development of a downlink beam training/alignment method for IRS-assisted mmWave/THz systems.	Specific	Specific
[38]	Secure transmission for an IRS-assisted mmWave and THz system.	Specific	Specific
[39]	Secure THz communication with IRS.	Specific	Specific
[40]	Security challenges affecting RIS-empowered 6G wireless networks.	Negligible	Negligible
[41]	Far-field path loss using physical optics techniques, considering an IRS in propagation.	Specific	Specific
[42]	Beamforming and information transfer technique based on spatial modulation of the LIS elements index.	Specific	Specific
[43]	THz RISs for 6G communication links.	Intermediary	Deep
[44]	RIS for wireless communications: an overview of hardware designs, channel models, and estimation techniques.	Deep	Deep
[45]	RIS for smart wireless environments: channel estimation, system design, and applications in 6G networks.	Deep	Deep
[46]	A survey of 6G wireless communications: emerging technologies.	Negligible	Negligible
[47]	Overview of THz-specific signal processing techniques for wireless communications, emphasizing ultra-mMIMO systems and RIS.	Intermediary	Deep
[48]	A tutorial on THz band localization for 6G communication systems.	Deep	Deep
[49]	Seven defining features of THz wireless systems: a fellowship of communication and sensing.	Deep	Intermediary
[50]	RIS-aided THz communications.	Specific	Specific
[51]	Near-field study of holographic IRS operating at THz frequency band.	Specific	Specific
[52]	Holographic RIS for application to mMIMO systems at THz band.	Specific	Specific
[53]	RIS-assisted THz systems.	Specific	Specific
[54]	THz communications employing supporting UAVs and IRS.	Specific	Specific
This work	A comprehensive survey on IS-aided THz wireless communication focusing on channel modeling.	Deep	Deep

**Table 2:** Comparison of recent research works regarding IS on THz wireless communication channel modeling in terms of performance evaluation, system model, design objective, and optimization technique.

Reference	Performance Evaluation	System Model	Design Objective	Optimization Techniques
[29]	Path gain and capacity	D–band indoor downlink	Maximize signal reflection and restore LoS link between the transmitter and receiver blocked by obstacles	Antenna theory
[30]	Adaptively selecting step size	Indoor MIMO downlink	Spectral efficiency with phase shift adjust	Taylor expansion aided gradient descent
[31]	Path loss	THz systems downlink	Expression that determines the optimal phase shift	Electromagnetic theory
[32]	Path loss	MIMO	Power gain and energy efficiency	Beamforming
[33]	Beam pattern and quantization error	mMIMO	Channel estimation and transmission solutions with hybrid beamforming architectures	Geometric channel model and IAP-SP for CSI acquisition.
[34]	NMSE	Indoor MIMO systems	Channel estimation	Beam training
[35]	NMSE	MIMO	Channel estimation	IAP-SP scheme
[36]	SNR feedback	MISO downlink	Minimization transmit power while maximizing the system achievable rate	Beamforming optimization based on statistical CSI and genetic algorithms
[37]	Success rate and beamforming gain ratio	mmWave/THz downlink	Perfectly aligned transmitter-receiver channel	Efficient beam training with cascade channel
[38]	Secrecy rate	mmWave/THz systems	Optimal discrete phase shifts to maximize the secrecy rate	SDP-based method and the element-wise BCD method
[39]	Secrecy rate	MISO downlink	Secure operation with secrecy rate maximization	Active beamformer and passive reflecting phase shifters
[41]	Path loss	Not detailed	Intensity for the electric field reflected by RISs in the short and long transmission distance regimes	General scalar theory of diffraction and the Huygens-Fresnel principle
[42]	SNR	Not detailed	Improve the average receive SNR	Beamforming
[50]	SNR, SNDR, small-scale amplitude fading, OP, and ergodic capacity	THz system	Exact PDF and CDF expressions of end-to-end SNR and SNDR of the system	FTR distribution and multivariate Fox’s H-function
[51]	Path loss and beam pattern	THz systems	Beamfocusing	Beamforming with physical optics channel model
[52]	Beam pattern	THz massive MIMO systems (downlink and uplink)	Beamforming design and channel estimation performance	Beamforming with physical channel model
[53]	SNR, ergodic capacity and OP	THz indoor systems downlink	Receiver beam misalignment and restore the transmitter/receiver connection blocked by obstacles	Electromagnetic theory
[54]	UAV trajectory, phase shift, THz band allocation and power control	UAV systems downlink	To maintain reliable THz communication and minimize average rate of users	SCA
[129]	Energy-efficient, SNR, latency and success rate	3-D indoor THz wireless communication	Minimizes latency using ray tracing techniques to find the best THz signal propagation path	Ray searching and beam-selecting
[130]	Achievable rate, phase compensation and normalized array gain	IS-aided THz system	Achievable rate maximization for distributed IS-assisted THz communications	Analog beamforming, digital beamforming vector
[131]	Phase shifts, ergodic net throughput, blocking probability	CF mMIMO	Channel estimation and unblock links	OP and fading spatial correlation with increased element numbers
[128]	Misalignment fading	THz inter-satellite links	Compensation for the high path loss associated with high carrier frequencies and to improve SNR	Antenna theory
[132]	Coverage area and sum rate	mmWave or THz indoor communication	Placement optimization to maximize the long-term sum rate and then optimization of transmit beamforming and reflecting procedure in real time	Antenna theory and deep learning
[133]	BER and modulation speed	Full-wave simulation	Eliminate on-chip signal attenuation and inter-symbol interference	Binary IS optimization algorithm

## Data Availability

Not applicable.
